# Can RNA Affect Memory Modulation? Implications for PTSD Understanding and Treatment

**DOI:** 10.3390/ijms241612908

**Published:** 2023-08-17

**Authors:** Tehila Cohen, Noam Shomron

**Affiliations:** 1 Faculty of Medicine, Tel Aviv University, Tel Aviv 6997801, Israel; 2 Edmond J Safra Center for Bioinformatics, Tel Aviv University, Tel Aviv 6997801, Israel; 3 Sagol School of Neuroscience, Tel Aviv University, Tel Aviv 6997801, Israel; 4 Tel Aviv University Innovation Labs (TILabs), Tel Aviv 6997801, Israel

**Keywords:** memory, transcription, RNA, microRNA, PTSD

## Abstract

Memories are a central aspect of our lives, but the mechanisms underlying their formation, consolidation, retrieval, and extinction remain poorly understood. In this review, we explore the molecular mechanisms of memory modulation and investigate the effects of RNA on these processes. Specifically, we examine the effects of time and location on gene expression alterations. We then discuss the potential for harnessing these alterations to modulate memories, particularly fear memories, to alleviate post-traumatic stress disorder (PTSD) symptoms. The current state of research suggests that transcriptional changes play a major role in memory modulation and targeting them through microRNAs may hold promise as a novel approach for treating memory-related disorders such as PTSD.

## 1. Introduction

Amid the chaos and violence of ancient battles, a different kind of casualty often went unnoticed. It was not a physical wound, but a psychological one, inflicted by the horrors of war on the minds of soldiers. In the earliest literature of mankind, we can glimpse the traces of this invisible damage, recorded in the stories and legends that have survived to this day. In the book of Deuteronomy, we find a passage that speaks to the psychological toll of warfare. It describes how military leaders would assess the courage of their troops before going into battle, asking: ‘What man is there that is fearful and fainthearted? Let him go and return unto his house, lest his brethren’s heart faint as well as his heart.’ Even in ancient times, military commanders recognized that witnessing death and experiencing the trauma of war could leave lasting scars on the minds of their troops. Thanks to the progress made in neuroscience and molecular biology over the past few decades, we now have a better understanding of the intricate mechanisms that underlie memory formation, storage, and maintenance. These complex processes involve numerous molecular actors, each playing a vital role in shaping our memories. One of these molecular actors is RNA, which has been the subject of extensive research since the 1980s. In particular, recent studies have shed light on the role of messenger RNA (mRNA) alterations in the formation and modulation of fear memories, which are often abnormally regulated in cases of post-traumatic stress disorder (PTSD).

### 1.1. Memory Modulation Stages in Various Brain Regions

Human memory is a mysterious and intricate system that allows us to hold on to our past experiences and shape our future. The study of memory modulation has made significant strides in understanding the various stages of memory formation, including acquisition, consolidation, retrieval, reconsolidation, and extinction, as well as the brain regions involved in these processes. PTSD is thought to be the result of an impaired fear memory modulation, where individuals with PTSD often have difficulty processing traumatic memories, leading to the development of intrusive and distressing memories, avoidance behaviors, and emotional dysregulation [1]. These symptoms are thought to be related to an inability to properly consolidate and store traumatic memories, leading to a heightened state of fear and emotional reactivity [2]. Fear memory is the most understood form of memory owing to methods such as fear conditioning, researched for more than 60 years. Other types of memories include spatial, contextual, working, and episodic memories. In this introduction, we refer to experiments carried out in humans, and if animal models were involved, we will mention it in each case [3].

Acquisition refers to the initial stage of memory formation, where new information is encoded and stored in the brain. Consolidation is the process by which the newly acquired information is stabilized and made more permanent.

Accessing stored information is referred to as retrieval and is a critical aspect of memory maintenance. After retrieval, memories can follow one of three paths: reconsolidation, when a consolidated memory becomes temporarily susceptible to change; extinction, when a memory is suppressed by conflicting information; or forgetting, when a memory loses significance over time [4]. Forgetting is a complex process that has been linked to several underlying mechanisms, including the formation of mature neurons from neural stem cells and the elimination of synapses by microglia [4].

The intricacies of memory modulation involve the participation of multiple brain regions, including the HPC, amygdala, neocortex, and cerebellum. The HPC plays a crucial role in acquiring and consolidating new memories, while the amygdala is involved in the emotional processing of memories. However, the traditional separation of emotional and cognitive brain regions is context-dependent, as they are deeply interwoven and influence each other in ways that contribute to adaptive and maladaptive behavior, thus challenging the conventional beliefs about the constituents of these regions [5]. The neocortex is responsible for the storage and retrieval of memories, and the cerebellum is involved in motor learning and memory. Functional magnetic resonance imaging (fMRI) studies suggest that similar brain regions are activated during retrieval and acquisition, though there may be some differences in molecular mechanisms [6]. Electrophysiological experiments in rodents, animals often used in research of memory due to their ease of maintenance and their molecular resemblance to humans, indicate bidirectional communication between the prefrontal cortex (PFC) and HPC during memory retrieval, with the PFC appearing to dictate which memories will be activated in the HPC [7]. Additionally, the medial prefrontal cortex (mPFC) is important in various phases of memory modulation, mainly formation and consolidation [8].

Interestingly, research suggests that there may be disruptions in the normal functioning of these brain regions in individuals with PTSD, particularly in the HPC and amygdala, leading to the development of persistent and distressing memories of the traumatic event [8,9].

### 1.2. Memory Modulation Associated Molecular Changes

Recent research in the field of memory has revealed new insights into the molecular mechanisms that govern memory formation and modulation. There have been extensive studies of the molecular mechanisms of acquisition, with much of the understanding coming from research on the intracellular signaling pathways activated by the N-methyl-D-aspartate (NMDA) receptor, a glutamate receptor playing a major role in synaptic plasticity [10]. Furthermore, the molecular mechanisms of consolidation also involve the replay of neural activity patterns that were established during training, which occurs at the system level [6].

One area of particular interest is the study of transcriptional changes in memory processes. The central dogma of gene expression involves the transfer of gene sequences from DNA to mRNA (transcription), and then to proteins (translation). The collective change in mRNA is referred to as the transcriptome, or all mRNA transcripts. Alterations in the expression of genes are thought to play key roles in many cellular activities, including in the formation and maintenance of memories. The changes in gene expression can occur in response to a variety of stimuli, such as learning, stress, or aging, and are thought to be crucial for the formation and modulation of memories. Gene expression changes can be measured by extracting RNA from the desired tissue, often brain regions in mice and peripheral blood in humans. Changes in RNA levels can then be analyzed by using methods such as microarrays, RNA sequencing (RNA seq), and quantitative PCR.

Furthermore, research has shown that regulatory RNAs, such as microRNAs (miRNAs) which bind mRNA and shut off their translation into protein, play an important role in the regulation of gene expression and memory modulation [11]. MicroRNA levels can be measured using similar methods to the transcriptome analysis methods mentioned above. To test the effects that microRNAs have on their gene targets, a variety of approaches could be used, including microRNA mimics, inhibitors, and mRNA target site blockers. Many studies have demonstrated that miRNAs can regulate the expression of genes involved in memory formation [11,12].

**Figure 1:**
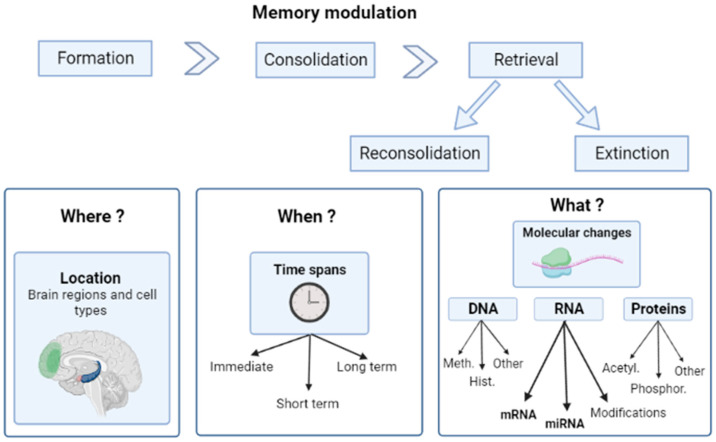
A scheme of the memory modulation flow. Memory modulation stages, divided into ‘Where’, ‘When’, and ‘What’, are the features. Left to right: The main brain regions and cell types involved in memory modulation and those that are mentioned in this review (in reference to PTSD): frontal cortex (green), hippocampus (blue), and amygdala (red). Time spans of memory modulation: immediate (hours), short-term (days and months), and long-term (years) effects. Molecular changes involved in memory modulation: mRNA represents the coding RNAs and miRNA (microRNA) represents the large group of non-coding RNAs (another group is long non-coding RNAs, or lncRNAs). Meth., DNA methylation; Hist., histone modification; Acetyl., protein acetylation; and Phosphor., protein phosphorylation. Created with BioRender.com, accessed on 23 May 2023.

It is worth noting that there are other types of changes that are involved in memory formation such as alterations in post-translational protein modifications (reviewed in [13,14]), epigenetic modifications (reviewed in [15]), and others. However, in this review, we will focus on the transcriptional changes (Figure 1).

### 1.3. Impaired Memory Modulation in PTSD

PTSD is a severe and debilitating mental health condition that can arise after exposure to a traumatic event. Its symptoms include intrusive and distressing memories of the traumatic event, avoidance behaviors, and emotional dysregulation. The persistent nature of traumatic memories is a defining feature of PTSD, and research has shown that this may be due to disruptions in normal processes of fear memory extinction and reconsolidation [2,16].

In individuals with PTSD, these processes may not work effectively, leading to the over-expression of fear responses and the maintenance of fear memories. Additionally, PTSD is associated with alterations in the function of brain regions involved in memory and emotional processing, such as the HPC, amygdala, and PFC [17]. These changes, along with the persistent and distressing nature of traumatic memories, can contribute to the development and maintenance of PTSD symptoms.

In this review, we will delve into five recent areas of interest that shed light on the transcriptional changes that affect memory processes. Specifically, we will explore: (i) the role of synaptic activity in memory modulation; (ii) the importance of specific time spans for transcriptional changes during different stages of memory modulation; (iii) the increased transcriptional activity in non-neuronal cells during memory consolidation; (iv) the potential for modulating mRNA changes to affect specific stages of memory modulation; and (v) the implications of such changes for fear memory modulation in PTSD.

## 2. Main

### 2.1. Synaptic Dynamics in the Memory Modulation Puzzle

Memory modulation is a complex and multifaceted process that involves intricate networks of brain regions, cells, and molecular pathways. At the heart of this process are synapses, which are crucial for brain plasticity and memory formation. We will first delve into the general role of synaptic activity in different stages of memory modulation, exploring how changes in synaptic strength and connectivity contribute to memory formation, consolidation, and retrieval. Second, we will examine the gene expression alterations that accompany these changes, shedding light on the complex molecular mechanisms that underlie memory modulation.

#### 2.1.1. Role of Synaptic Activity in Memory

Synaptic activity plays a crucial role in memory modulation by facilitating the formation and consolidation of memories. The process of memory consolidation involves the transformation of a short-term memory into a long-term memory through the stabilization and strengthening of synaptic connections [18]. This process is known to be dependent on the activation of NMDA receptors, which leads to the activation of intracellular signaling pathways and gene expression that ultimately result in synaptic plasticity [19]. Additionally, synaptic activity can modulate memory by regulating the encoding and retrieval of memories [20]. During encoding, synaptic plasticity allows for the formation of unique and stable connections between neurons that encode a specific memory [21]. During retrieval, the reactivation of these connections through synaptic activity is necessary to access and retrieve the stored memory [21]. Furthermore, synaptic activity is involved in the process of fear memory extinction, where the association between a previously learned cue and its aversive response is weakened [22]. The weakening of this association is thought to occur through the modification of synaptic connections, which ultimately leads to the attenuation of the fear response. To develop effective strategies for targeting memory disorders, it is crucial to understand the molecular mechanisms that modulate memory through synaptic activity. Such an understanding is essential for identifying ways to manipulate these mechanisms and improve memory function in individuals with memory disorders.

#### 2.1.2. Synaptic Gene Expression and Its Effects on the Stages of Memory Modulation

Previous research on the formation of long-term memories has shown that this process requires changes in the transcription of specific genes at the synapse. The transcription factor CREB and several modulators, including CREB-regulated transcription coactivator-1 (CRTC1), histone deacetylase 4 (HDAC4), and nuclear factor kappa-light-chain-enhancer of activated B cells (NF-κB), were found to play critical roles in this process. Genetic enhancement of Creb and Crtc1 activity in the forebrain and HPC of mice, respectively, increases fear memory, while knockdown of Crtc1 leads to decreased fear memory and CREB-mediated gene transcription (Table 1) [23,24,25,26]. Interestingly, bioinformatic analysis of experimentally validated database of human transcriptomes [27], suggests that miR-34a-5p may regulate the expression of CRTC1 in brain tissue, thus reducing the gene’s expression. It is possible that other miRNAs regulate CRTC1 expression, among other genes related to memory processes. Therefore, the regulation of CRTC1 by miRNAs represents a potential mechanism for modulating fear memory in mice.

Another key player in memory formation is neuronal PAS domain protein 4 (NPAS4). NPAS4 is a transcription factor that plays a crucial role in the formation of contextual memory by inducing the transcription of genes in the cornu ammonis 3 (CA3) region of the HPC [28]. It was previously shown that both global and CA3-specific knockout of the Npas4 gene in mice impaired contextual fear conditioning [29]. This memory deficit was later corrected with selective expression of Npas4 in the CA3 of the HPC [29]. This study and many others showed that Npas4 is essential for the expression of activity-dependent genes during inhibitory synapse development, and its transcriptional program might shape feedforward inhibition in the HPC, refining the context of memory [28].

Memory consolidation relies on intricate mechanisms of synaptic activity, too. Recent research has identified a highly connected network of genes involved in vesicle-mediated transport, exocytosis, and neurotransmitter secretion that are crucial for remote-memory-dependent transcriptional programs [30]. A study in mice brains found that a significant number of remote-memory-associated differentially expressed genes (DEGs) were related to these functional categories, including syntaxin-1B (STX1B), synaptotagmin-13 (SYT13), vesicle associated membrane protein 2 (VAMP2), N-Ethylmaleimide sensitive factor (NSF), and RAB5A, all linked to the SNARE complex and vesicle exocytosis [30]. Serine incorporator 1 (SERINC1) and serine incorporator 3 (SERINC3) were the most highly upregulated genes, potentially promoting vesicle membrane fusion during memory consolidation in mice [30]. Experiments using activity-dependent labeling of neurons and single-cell transcriptomics revealed additional evidence for this process [30]. These results strengthen the notion that enhanced membrane fusion and vesicle exocytosis may be critical modes of synaptic strengthening during memory consolidation. More specifically, they suggest that a specific set of exocytosis-related genes may be involved in facilitating the formation of highly unique, experience-specific connections. These transcriptional programs appear to be detectable at remote time points, hinting at their possible role in maintaining the memory trace weeks after learning. Thus, these findings indicate that synaptic transcriptional activity plays a key role in memory modulation, shedding light on the molecular mechanisms that could be used to modulate specific memory consolidation [30].

Recently published research on synaptic molecular mechanisms of memory consolidation revealed new insights into the role of KH-type splicing regulatory protein (KHSRP) [31]. Specifically, the loss of KHSRP was found to increase neuronal growth and synaptic transmission while also altering memory consolidation through RNA stabilization [31]. The splicing-regulating protein KHSRP plays a crucial post-transcriptional role in regulating the expression of several target mRNAs involved in neuronal development and function [31]. Mice lacking Khsrp (Khsrp^−/−^) showed alterations in neuronal morphology and function, including increased axon and dendrite growth, and elevated levels of Khsrp-targetted mRNAs [31]. Importantly, the loss of Khsrp has also been shown to impair memory consolidation in both the PFC and HPC, demonstrating the critical role of this protein in regulating the processes underlying normal brain function. Interestingly, using an experimentally validated database [27], we found that KHSRP expression can be modulated by miRNAs such as miR-98-5p (among others, see Table 2) in human brain tissues. This targeted method of regulating KHSRP expression presents a potentially useful strategy for modulating memory consolidation. Thus, these findings provide insight into the intricate mechanisms by which KHSRP controls neuronal development and function in memory consolidation through post-transcriptional regulation of gene expression [31].

Memory recall has also been the center of attention recently for its potential role in memory modification and extinction. According to findings from studies on fear conditioning and recall in mice, fear-associated DEGs were found to be non-overlapping with remote-memory-associated DEGs, indicating that the experience of fear may induce different long-lasting changes in gene expression programs. Furthermore, the recall process appears to induce new transcriptional programs in a separate group of neurons, suggesting a complex interplay between different brain regions and gene expression patterns in the formation and maintenance of remote memories [30]. Other transcription factors, such as firkhead box p1 (FOXP1), serum response factor (SRF), and myocyte enhancer factor 2 (MEF2), also affect memory modulation through synaptic activity modulation. Foxp1 knockout impairs spatial memory, while Srf knockdown in the forebrain impairs the formation of immediate memory in response to a novel context [32,33]. Foxp1 expression can be regulated by miR-6951-3p which could be used to specifically modulate spatial memory in mice [32]. Neuronal overexpression of Mef2 restricts dendritic spine growth, impairing spatial and fear memory formation, while decreasing Mef2 levels in the dentate gyrus and amygdala improves memory formation [34]. Counterbalancing the negative effect of MEF2 by interfering with AMPA receptor endocytosis improves memory formation [30].

To conclude, the complex mechanisms involved in memory modulation through synaptic activity have been the subject of intensive research. Molecular players such as the genes CREB, CRTC1, HDAC4, NF-κB, and Npas4 were found to play key roles in memory formation and consolidation (Table 1). Moreover, recent studies identified a highly connected network of genes involved in vesicle-mediated transport, exocytosis, and neurotransmitter secretion that are crucial for remote-memory-dependent transcriptional programs. KHSRP was also identified as an important splicing-regulating protein that controls neuronal development and function through post-transcriptional regulation of gene expression. Furthermore, recall of memories appears to induce new transcriptional programs in a separate group of neurons, indicating a complex interplay between different brain regions and gene expression patterns in the formation and maintenance of remote memories. This extensive evidence surely broadens our understanding of memory processes, but there still remains an important question to be asked: when do these molecular changes happen?

### 2.2. The Importance of Transcriptional Changes at Specific Times of Memory Modulation

Prior research has already established that gene transcription and protein synthesis are critical for both consolidation and reconsolidation of long-term memories immediately following training or mnemonic trace reactivation [35,36]. Additionally, some studies have suggested that the consolidation of long-term memories may also require a second wave of gene transcription occurring later. For examples, Cláudio da Silva and Sartori Bonini [37] raised the question of whether a similar gene transcription exists for reconsolidation as well; when they blocked late gene expression during a specific time window after training or memory retrieval, long-term spatial memory retention was found to be impaired in the Morris water maze task. The results suggest that the transcription of late genes is necessary for the reconsolidation process in the HPC to stabilize the reactivated mnemonic trace. Interestingly, the transcription inhibitors impaired memory retention only when infused into the dorsal HPC 6 h, but not 9 h, after training [37]. This study also showed that late gene transcription was critical for spatial memory consolidation and reconsolidation in mice. This sensitivity was observed in a time window that began 3 h after training or retrieval and lasted less than 9 h. It should be noted that the inhibition of late gene transcription impaired long-term memory only if the retrieval occurred without reinforcement, and the result was structure-dependent, occurring only in the dorsal HPC [37]. Thus, when the mice’s memories were reinforced, the reduction in late gene transcription did not affect their long-term memory, perhaps because the reinforcement re-strengthened said memory in their brain. Additionally, the fact that this effect was localized in the dorsal HPC suggests that this is a critical area for long term memory retention.

A recent study investigated the role of de novo gene transcription in the reconsolidation of alcohol-associated memories in mice [38], thus emphasizing the importance of the sensitivity of memory processes to transcriptional programs at specific times in the process. In their study, Goltseker et al. showed that the reconsolidation of alcohol-associated memories requires de novo gene transcription, and that inhibiting said transcription immediately following alcohol memory retrieval disrupts alcohol seeking. This research suggests that the altered expression of certain genes is likely a common mechanism for the reconsolidation of several types of memories, but the processing of alcohol-related memories is further characterized by a unique transcriptional profile. The study found that the retrieval of either alcohol- or sucrose-related memories triggered similar increases in mRNA expression of the genes Arc and Egr1, both related to synaptic plasticity functions [38]. However, a subset of genes, including Adcy8, Slc8a3, and Neto1, were altered selectively by the retrieval of alcohol, suggesting the likelihood that alcohol-associated memories triggering relapse have unique molecular mechanisms that could be targeted to disrupt them selectively. The study shows that the downregulation of Arc shortly after alcohol memory retrieval disrupts alcohol seeking. These findings suggest a critical role for hippocampal Arc expression in the reconsolidation of alcohol memories. The research also revealed that the “reconsolidation window”, i.e., the timeframe in which memories can be altered, might be narrower than five hours when ARC protein expression upregulation is required for memory reconsolidation. The study suggests that the mPFC is a candidate brain region that regulates alcohol seeking through reconsolidation pathways, given its upregulated ARC protein levels following alcohol memory recall. Finally, the study indicates that the increases in the immediate early genes (IEGs) expression are not unique to the reconsolidation of alcohol-related memories. Instead, ARC and EGR1 were previously implicated in the reconsolidation of several types of memories, including fear memories, recognition memories, and memories associated with different drugs of abuse [38].

Taken together, the studies described above suggest a critical relationship between the timing and specificity of transcription in memory consolidation and reconsolidation. Moreover, they emphasize the importance of transcriptional time spans for memory processing and suggest that different types of memories may have unique transcriptional programs (Table 1). Thus, future research aimed at uncovering the molecular mechanisms underlying the transcriptional regulation of specific types of memories may provide novel therapeutic targets for memory-related disorders.

To this point we focused on neurons; could there be other cells involved?

### 2.3. The Transcriptional Activity beyond Neurons

The process of memory modulation is a complex and multi-faceted phenomenon, which involves not only the intricate network of neurons, but also the contribution of non-neuronal cells such as astrocytes and glia cells. Despite the significance of non-neuronal cells, they have often been overlooked in the study of memory modulation. However, recent studies have suggested that these cells play a vital role in many processes, including memory. To fully comprehend the mechanisms of memory modulation and to develop methods to manipulate them, it is essential to understand the role of every player involved.

Recent research has suggested that astrocytes may play a significant role in modulating higher cognitive function, such as working memory (WM), by influencing synaptic transmission. One study employed a computational model to investigate the impact of astrocytes on the stability and duration of working memories [39]. Their results indicated that the duration of working memory representations can be influenced by an astrocytic time constant, which defines a “window of vulnerability” during which some memories are tagged for long-term retention while others are terminated. The proportion of memories in the retention and termination groups can be regulated by adjusting the strength of astrocytic feedback or its time constant. These findings suggest that astrocytic signaling may serve as a candidate mechanism for top-down control of working memory representations and their duration [39].

Linking this to transcriptional activity, it is worth noting that non-neuronal cells also exhibit changes in transcriptional activity associated with remote memory consolidation. Chen et al. recently showed that non-neuronal cells’ transcriptional activity is distinct from those of neurons, indicating that non-neuronal programs may support the maintenance of the remote fear-memory trace [30]. Interestingly, they found that more than 95% of the DEGs were upregulated, suggesting an overall transcriptional activation during consolidation. It is also worth noting that the consolidation of remote memory induces a persistent transcriptional program in astrocytes and microglia. These findings are in line with previous research in Drosophila, which has shown that long-term memory formation requires increases in glial gene expression [40]. Together, these observations highlight the role of non-neuronal cells in memory consolidation and suggest that transcriptional changes in these cells may play a crucial role in the formation and maintenance of long-term memory.

In conclusion, the study of memory modulation involves a complex interplay between neurons and non-neuronal cells. While non-neuronal cells have often been overlooked in the past, recent research has highlighted their significant contributions to memory modulation, including the regulation of working memory representations and the transcriptional changes associated with remote memory consolidation (Table 1). These findings suggest that a comprehensive understanding of memory modulation requires a consideration of all actors involved, including non-neuronal cells, and their underlying mechanisms. By further exploring the role of non-neuronal cells and their transcriptional activity in memory modulation, researchers may pave the way for novel therapeutic interventions and ultimately enhance our ability to manipulate memory processes. Now that many molecular changes were identified and were shown to be involved in memory modulation, how can we make use of it?

### 2.4. Tuning Transcription for Enhanced Memory Control

Memory formation and consolidation are complex processes that rely on the coordinated regulation of numerous gene expressions. Over the past few years, research has made significant strides in elucidating the molecular mechanisms underlying memory formation and storage. In particular, studies have shown that changes in gene expression play a critical role in shaping various aspects of memory, from acquisition to consolidation to reconsolidation. As mentioned earlier, manipulating the expression of certain genes can have profound effects on memory processes. However, the question remains: can we harness this knowledge to fine-tune transcription and actively modulate memory processes in a more targeted manner?

Research has begun to explore the potential of transcriptional regulation as a tool for modulating memory processes. By manipulating the expression of key genes involved in memory formation and storage, such as through the use of miRNAs, the hope is to develop new strategies for enhancing cognitive function and treating memory-related disorders. Ultimately, our goal is to shed light on the exciting possibilities that lie ahead for harnessing the power of gene expression to regulate memory processes and more specifically fear memory pathways.

To investigate the impact of transcriptional regulation on memory processes, Penke et al. utilized an inducible transgenic mouse model to selectively induce Zif268 overexpression in forebrain neurons [41]. Through a series of experiments, they examined the effect of this manipulation on recognition memory and hippocampal synaptic transmission and plasticity. Interestingly, they found that Zif268 overexpression during the formation of memory for objects did not affect the ability to form a long-term memory of objects, but instead enhanced the capacity to form a long-term memory of the spatial location of objects. This enhancement was paralleled by increased long-term potentiation in the dentate gyrus of the HPC, as well as by increased activity-dependent expression of Zif268 and selected Zif268 target genes [41]. These findings suggest that targeted gene expression regulation has the potential to selectively modulate, and in this case enhance, different aspects of memory processes.

In 1998, genes that limit the ability to consolidate memories were identified; they were called “memory suppressor genes” [42]. The first ones to be discovered were thought to limit the activity of cAMP-dependent consolidation. Recently, more have been identified. Memory suppressor genes, now also known as memory repressor genes, play a crucial role in limiting the formation and expression of memories [42]. These genes have been found to affect not only consolidation but also acquisition and forgetting. This suggests that there may be a biological need to limit memory formation [43]. While most rodent learning paradigms measure memory performance hours to weeks after training, studies in Drosophila have identified several miRNAs that act as memory suppressor genes by inhibiting memory acquisition [43]. Additionally, consolidation suppressors prevent the encoding of inconsequential information into long-term memory. Finally, forgetting plays a critical role in behavioral flexibility and memory generalization. The identification of memory suppressor genes has provided valuable insights into the molecular mechanisms that underlie this important process. In this study, our focus will be on the “memory suppressor genes” that are associated with each stage of memory modulation.

#### 2.4.1. Memory Acquisition Suppressor Genes

The molecules of GABAergic inhibitory systems, which are encoded by certain genes, act as suppressors of acquisition [44]. The gamma-aminobutyric acid type A (GABAA) receptors, which are activated by the neurotransmitter GABA, function as ligand-gated chloride channels. These channels reduce the ability of neurons to depolarize and fire action potentials, thereby suppressing the acquisition of new memories [45]. This suppression occurs through various mechanisms, including the inhibition of the neural representation of stimulus strength, miR-980-mediated inhibition of the A2bp1 gene, regulation of gene expression by stromalin, and reduction in neurotransmitter persistence in the synapse by the transporter SLC22A [46]. Specifically, miR-980 limits memory acquisition in mice by decreasing the excitability of neurons. A2bp1 has been identified as a critical mediator of this effect [46]. Interestingly, the effects observed on neuronal excitability by miR-980 are similar to those observed when GABA receptor function is reduced [43]. In summary, suppressors of acquisition work by limiting the potency of stimuli, capping neuronal excitability, limiting neurotransmitter availability or release, and limiting neurotransmitter persistence/function in the synapse [43]. Further research is needed to gain a better understanding of the role of miR-980 in impairing memory acquisition.

#### 2.4.2. Memory Consolidation Suppressor Genes

A study has identified a suppressor of consolidation associated with CREB function in Aplysia [47]. Neutralizing antibodies to Aplysia CREB2, an endogenous CREB inhibitor, when injected into sensory neurons, allow a single pulse of serotonin to produce long-term changes in plasticity. These changes are typically not strong enough to drive such alterations [47]. Inhibition of activating transcription factor 4 (ATF4), a homolog of Aplysia CREB2 in mice, enhances spatial memory and long-term potentiation (LTP) [48]. It has been well established that CREB-dependent transcription is necessary for long-term memory consolidation. Additionally, it has been found that suppressors of CREB hinder the process of consolidating short-term memory into long-term memory. Several studies have examined the mechanism by which CREB promotes consolidation and how suppressing CREB prevents it. Some of these studies were discussed in previous sections of this review. CREB has been shown to modulate basal neuronal excitability and the development of LTP. Mice lacking CREB display impaired fear and spatial long-term memory, as well as a failure to develop hippocampal LTP [49]. On the other hand, overexpression of CREB increases neuronal excitability and long-term memory following suboptimal fear conditioning. In addition, studies suggest that PIWIL1 and PIWIL2 function as redundant memory suppressors in the mouse hippocampus, while miR-182 has been identified as a possible consolidation suppressor in the amygdala [50]. Interestingly, CREB1 mRNA levels can be regulated by miR-182-5p in human liver tissue but also by miR-338-3p and miR-495-3p in human brain tissue. Through this targeted process, memory consolidation can be modulated and studied [51]. These findings emphasize the significance of CREB-dependent transcription, neuronal excitability, and miRNA regulation in the consolidation of long-term memories and potentially in its modulation.

#### 2.4.3. Memory Extinction Suppressor Genes

The strength of synaptic connections can be modulated by changes in the levels of α-amino-3-hydroxy-5-methyl-4-isoxazolepropionic acid receptor (AMPAR), which play a crucial role in memory formation and forgetting [52]. After learning, the insertion of additional AMPARs into postsynaptic sites increases synaptic strength. Conversely, the internalization of surface AMPARs reduces the functional connection between potentiated synapses and is considered one mechanism for forgetting [53]. The GLU2A subunit of AMPAR in the mouse amygdala has been found to have a positive correlation with memory strength after fear conditioning. Additionally, inhibiting the internalization of hippocampal Glu2a following training suppresses the forgetting of episodic memory [54,55]. Two genes, CASPASE-2 and SYT3, have been identified as regulators of memory stability through the internalization of AMPARs [56,57]. The general principles learned to date indicate that memory suppressor genes encode molecules that participate in the endocytosis of neurotransmitter receptors. This process helps to reduce excitatory synaptic input. By activating specific dopamine receptors on postsynaptic engram cells, certain dopamine neurons can trigger a signaling cascade. This cascade leads to the activation of small G-proteins and the rearrangement of the cytoskeleton in those cells. Ultimately, this process results in the removal of memories. These mechanisms offer attractive options for reducing the receptive state of engram cells or modifying the structural changes at synapses that occur during memory formation and consolidation. By doing so, they can enhance targeted forgetting.

In conclusion, experiments have shown that changes in gene expression play a critical role in shaping various aspects of memory, from acquisition to consolidation to reconsolidation (Table 1). The manipulation of the expression of specific genes has profound effects on memory processes. The emerging field of transcriptional regulation and memory control has explored the potential of targeted transcriptional regulation to selectively modulate different aspects of memory processes. Memory suppressor genes have been identified as playing a crucial role in limiting the formation and expression of memories. Harnessing the power of gene expression to regulate memory processes, particularly fear memory pathways, presents exciting possibilities for enhancing cognitive function and addressing memory-related disorders, but what are the implications for PTSD?

### 2.5. The Path to Fear Memory Extinction through Molecular Targets in PTSD

As previously reviewed, the process of memory modulation is complex and involves various transcriptional changes that occur at different stages. Understanding these changes is crucial for exploring their potential implications in the context of PTSD. This mental health condition is characterized by impaired modulation of fear memory, which can have debilitating consequences for those affected by it. By leveraging our knowledge of transcriptional changes in memory modulation, we can identify potential strategies to modify this process and alleviate symptoms of PTSD.

Recent studies have begun to shed light on the underlying molecular mechanisms that drive the formation and modulation of memories in PTSD, including on the transcriptional changes that occur in response to traumatic events. Studies of peripheral blood of PTSD patients have shown alterations in the expression of genes that are thought to play a key role in the formation and maintenance of memories [58]. For example, the expression of genes such as FK506-binding protein 5 (*FKBP5*) and *BDNF* are altered in PTSD patients in the brain and in the peripheral blood, respectively. FKBP5 and BDNF are related to the regulation of stress response and neural plasticity, respectively, and the study showed that changes in these genes’ expression levels may contribute to the development and maintenance of PTSD symptoms [59]. Additionally, other previously mentioned experiments indicated that the expression of genes such as protein kinase A (*PKA*) and *CREB*, linked to synaptic plasticity and memory consolidation, are altered in PTSD patients [60].

Before delving into the transcriptional regulation of memory, particularly fear memory in PTSD, it is important to recognize the crucial role of animal models in uncovering the foundations of most findings in this field. Memory modulation research benefits from the synergy among animal models, clinical studies, and in vitro models. Animal models, especially rodents, offer invaluable insights into the intricate processes of memory formation and modulation. By investigating specific molecular changes, such as alterations in mRNA and microRNA expression, at different stages of memory modulation and within various brain regions, these models provide a comprehensive understanding of the molecular dynamics at play. Moreover, animal models offer distinct advantages for studying memory-related disorders such as PTSD. They provide a level of experimental control that is difficult to achieve in human studies. Furthermore, animal models provide a platform to examine the effects of potential therapeutic interventions. It is important to exercise caution when extrapolating findings from animal models to human conditions. However, these models play a critical role in generating hypotheses and designing subsequent studies involving human subjects. In the context of PTSD, animal models often use stress paradigms that involve single acute stressors such as physical, predator, or social stress. These preclinical models mimic PTSD phenotypes and are assessed using various behavioral tests that measure the expression of PTSD-like symptoms. In summary, the use of animal models in memory modulation research, including fear memory in PTSD, is essential for deciphering the molecular basis of these processes. The controlled experimental conditions, along with behavioral assessments, provide a valuable foundation for investigating the transcriptional changes associated with memory modulation and offer insights into the complexities of PTSD.

The effects of ketamine on fear memory extinction were investigated [61]. The research team examined changes in glutamate transmission and dendritic arborization in the PFC of rats subjected to footshock-induced stress. The study found that ketamine administration blocked the acute stress-induced enhancement of glutamate release. This effect was observed 24 or 72 h before or 6 h after the footshock. Furthermore, ketamine administration rescued the retraction of apical dendrites in pyramidal neurons and facilitated the extinction of contextual fear. The study revealed that ketamine has a rapid effect on animals subjected to acute stress. This suggests that there is a mechanism at play involving the restoration of glutamate release and structural remodeling of dendrites. The acute ketamine administration stabilized the dysfunctional release of glutamate induced by stress in animals. The study highlighted that the acute ketamine administration exerted different effects on PFC glutamate release in naïve rats compared to animals subjected to acute foot shock stress [61]. These findings suggest a potential mechanism of action for the rapid antidepressant effect of ketamine, which involved the re-establishment of synaptic homeostasis in the PFC. Overall, this work offers valuable insights into the immediate effects of ketamine in animals subjected to acute stress. These findings could have significant implications for the development of novel therapeutic strategies for stress-related psychiatric disorders. To date, no study has specifically investigated the transcriptional changes associated with ketamine treatment response in PTSD. However, a study did examine the changes in transcriptional signatures in response to ketamine treatment in depression and focused on the role of glutamate in this process [62]. Before administering ketamine, the study identified that two genes associated with glutamate signaling, GRM2 and GRIN2D, were more abundant in ketamine responders compared to non-responders [62]. In line with the findings from the ketamine study in PTSD, these suggest that glutamate receptors may be involved in the response to ketamine. Thus, targeting GRM2 could present a potential mechanism for modulating stress-related disorders.

Another established target is modulating the expression of the BDNF gene as a therapeutic approach for fear memory extinction. Impaired fear extinction is a hallmark of PTSD, and BDNF appears to enhance this process [59]. A study focused on the role of BDNF on fear memory extinction. BDNF is necessary for the formation of emotional memories in areas of the brain such as the amygdala, HPC, and PFC. Different systems, such as the endocannabinoid system and the hypothalamic–pituitary–adrenal axis, modulate fear extinction through BDNF [59]. Exogenous fear extinction enhancers, such as antidepressants and N-methyl D-aspartate agonists, may act through or in concert with the BDNF-TrkB system [63]. Genetic manipulations of BDNF and TrkB in mice demonstrate that BDNF and TrkB are essential for CNS development and play a key role in synaptic plasticity and the formation of fear memories [64]. Furthermore, there are interesting interactions between the endocannabinoid system and BDNF/TrkB, which would be important for the modulation of fear extinction [65]. The generation of conditional knockout mice has shown that fear extinction effects are regionally dependent. While BDNF deletion in the HPC leads to cue-dependent fear extinction deficits, no effect is found in extinction of cue-dependent fear when the BDNF deletion is restricted to the prelimbic cortex (PL). However, BDNF in the PL is necessary for cue-dependent fear acquisition, and the deficit in learned fear is rescued by 7,8-DHF, which mimics endogenous BDNF by activating TrkB receptors [59]. Additionally, in our own analysis we identified several miRNAs that can potentially regulate BDNF expression, such as miR-30d-5p human brain tissue (refer to Table 2 for the exhaustive list) [66]. Furthermore, a distinct set of miRNAs linked to the modulation of BDNF expression exhibited varying regulation patterns in mice predisposed to developing PTSD-like behaviors in contrast to resilient mice [67]. These specific miRNAs revealed the potential to distinguish between the contrasting stress-related phenotypes observed in mice. The variations in miRNA expression might play a role in influencing the inversely related transcript levels of BDNF [67]. These observations show that targeting the regulation of BDNF expression by those miRNAs may be a potential target for regulating fear extinction in individuals with PTSD.

Memories are not static entities but rather constantly changing and updating over time. This makes them susceptible to modulation or alteration, which has led research to explore various psychological interventions to modulate memories. One of the main strategies involves targeting the unstable state of memories after retrieval, which can make them more malleable and susceptible to modification. In this approach, memories are temporarily destabilized through retrieval and then modified through various interventions, such as exposure therapy or cognitive reappraisal. This technique has shown promise in treating a range of psychological disorders, including anxiety disorders, PTSD, and addiction. To better grasp the molecular aspect of this phenomenon, a study investigated the mechanisms involved in fear memory reconsolidation and extinction in mice and suggested a transcription factor switch that would determine the memory course after retrieval [68]. The authors found that two transcription factors, NF-κB and NFAT, have opposing roles in these processes. NF-κB is required for fear memory reconsolidation, while calcineurin (CaN) phosphatase inhibits NF-κB and induces NFAT nuclear translocation, promoting memory extinction. The inhibition of both calcineurin and NFAT impairs memory extinction, while inhibition of NF-κB enhances memory extinction [68]. Interestingly, a study uncovered a noteworthy connection: through the inhibition of miR-142, a pivotal regulator of the fragile mental retardation protein (FMRP), they were able to impede the translocation of NF-κB into neuronal nuclei. This potentially had implications for the shape and structure of neurons. This discovery was part of a broader investigation where the suppression of miR-142 was observed to alleviate PTSD-like behaviors in rats subjected to a stress paradigm. This insight further solidified the link between NF-κB and the regulation of fear memory [69]. Additionally, the modulation of PTSD-like behavior in rats following a stress paradigm has been demonstrated by regulating FMRP through other miRNAs, such as miR-132 [70].

The report on the transcription factors, NF-κB and NFAT, describes two transcriptional mechanisms that are differentially induced in either reconsolidation or extinction in associative learning. NF-κB is activated in the HPC and is necessary for memory reconsolidation, whereas hippocampal CaN activity is necessary for extinction. Inhibition of both CaN and NFAT in the hippocampus impairs extinction but does not affect reconsolidation. One key downstream target for NFAT in the nucleus is the BDNF promoter [68]. This finding is consistent with the previously mentioned study that discusses the role of BDNF on memory extinction, as it has demonstrated that BDNF plays a crucial role in enhancing fear memory extinction. The findings suggest that a restriction of the transcription factor NF-κB is part of the mechanism involved in extinction. Overall, this research sheds light on the intricate and opposing roles of transcription factors in fear memory reconsolidation and extinction. The findings highlight the importance of NF-κB in memory reconsolidation and the inhibitory effects of calcineurin and NFAT in promoting memory extinction. The implications of these findings for the development of therapeutic strategies for fear memory disorders are significant, and it brings us one step closer to understanding how we can potentially manipulate them to alleviate related disorders.

Many of the studies mentioned in this review have shown that memory modulation is associated with transcriptional changes in the brain (Table 1). However, the question that arises is which symptoms exactly those transcriptional changes have effects on. To better understand how to modulate memories for PTSD, it is important to identify the specific symptoms that these transcriptional changes help alleviate and those that they do not. This knowledge can inform the development of more effective and targeted therapies for PTSD. Interestingly, a relatively recent study focused on the gene expression differences attributed to different PTSD symptoms clusters [71]. Using a transcriptome-wide analysis of differential gene expression in peripheral blood, the researchers found that gene expression differences between individuals with PTSD and control participants were mainly attributed to the intrusion symptom cluster. Specifically, ten genes were upregulated in participants with PTSD and high intrusion symptoms at baseline, and interestingly, were downregulated in participants with improved PTSD symptoms following treatment. Among these genes, RBAK, a DNA transcription regulator, was the top upregulated gene associated with PTSD, while C5orf24 was the most downregulated gene associated with symptom improvement [71]. Taken together, these findings suggest that specific molecular biomarkers may inform the development of targeted therapies for the precise treatment of PTSD, particularly for individuals with high intrusion symptoms.

Our preceding discussion unveiled the intricate landscape of gene expression related to memory, highlighting time- and memory-dependent changes in transcription that influence memory modulation. Another highly important aspect to consider are the sex-dependent differences, which are particularly significant given the elevated vulnerability of females to PTSD. However, preclinical research has predominantly focused on male rodents. To illuminate stress’s impact on recognition memory, a study investigated sex-dependent shifts in the transcriptional changes of ionotropic glutamate receptor subunits following stress, revealing altered expression of NMDA and AMPA receptor subunits [72]. Another investigation delved into the sex-dependent role of the methyl-CpG binding protein 2 (MECP2). Through examining MECP2 mRNA levels in a cohort of 132 participants, including 58 women, a compelling narrative emerged. Among women exposed to trauma and adverse childhood experiences, the downregulation of MECP2 correlated with heightened PTSD symptoms [73]. This underscores MECP2′s role in post-trauma pathophysiology and calls for further investigation into its sex-specific implications for the onset and progression of PTSD.

Transcriptional changes are not the only ones prone to sex-dependent influences. Within the realm of epigenetic regulation, the histone variant H2A.Z has emerged as a potent controller of fear memory suppression [74]. By utilizing mice with a conditionally inducible H2A.Z knockout (cKO), researchers unveiled nuanced sex-dependent dynamics, revealing a situation where male-specific enhancement of fear memory occurred [74]. Notably, H2A.Z cKO elevated non-stress-related memory in both genders, underscoring its dual impact regardless of sex. Due to the distinct vulnerability of females to PTSD, the focus turned towards examining the effects of H2A.Z cKO in a stress-amplified fear learning model. Encouragingly, this manipulation selectively mitigated fear sensitization and pain responses in females, untangling the interconnected influences of sex, task type, and stress history. Consequently, H2A.Z emerges as a potential sex-specific epigenetic factor in PTSD susceptibility, shedding light on avenues for personalized therapeutic strategies. These findings emphasize the importance of integrating gender-specific mechanisms into conversations about stress-induced memory alterations, a pivotal aspect when crafting tailored intervention approaches.

When investigating miRNAs regulation in PTSD in this review, we primarily focused on those that regulate the expression of key genes associated with general memory and fear memory modulation. Nevertheless, research also indicates the involvement of various miRNAs directly on PTSD susceptibility, symptomatology and the regulation of fear memory. For example, a study focused on investigating the impact of stress and stress-enhanced fear learning on the mir-598-3p levels. Interestingly, the study uncovered that this miRNA shows increased levels in the basolateral amygdala (BLA) of male mice, who are susceptible to the effects of stress. However, this response was not observed in female mice. Intriguingly, when they inhibited mir-598-3p within the BLA, they noted disruption in the expression and extinction of remote fear memory, although this effect was statistically significant only in male mice. Overall, the outcomes of their investigation imply that stress-induced activation of BLA mir-598-3p might play a crucial role in orchestrating a shift from a typical remote fear memory to one that is intensified and resistant to extinction, and that this phenomenon might be sex-dependent [75]. Others have revealed the potential of miR-153-3p in alleviating PTSD symptoms. They showed that miR-153-3p levels were upregulated in the hippocampus of rats subjected to a single prolonged stress paradigm. The attenuation of miR-153-3p led to a reduction in PTSD-like behaviors among the rats exposed to the stress paradigm [76].

In conclusion, fear memory modulation is a complex process that involves various transcriptional changes occurring at different stages, which are crucial to explore their potential implications in the context of PTSD. Studies have investigated potential avenues to tune this process and alleviate PTSD symptoms by identifying molecular targets for fear memory extinction. Recent studies on ketamine, BDNF regulation, specific transcription factors, miRNAs, and changes in gene expression have all highlighted promising avenues for improving fear memory modulation. These findings suggest that combining these approaches may offer a promising avenue for developing effective therapies for anxiety and/or memory disorders characterized by impaired fear memory extinction. Targeting the unstable state of memories after retrieval is another approach that has shown promise in treating a range of psychological disorders, including PTSD. Overall, these approaches offer hope for developing effective treatments for those affected by PTSD and other mental health conditions characterized by impaired fear memory modulation.

**Table 1:** Summary of mRNA changes mentioned in this report and their effects on memory modulation. HPC = hippocampus, DG = dentate gyrus, and CA1, CA2, and CA3 are different parts of the hippocampus circuit.

mRNA	Organism	Brain Region	Regulation	Effect	Reference
*Creb*	Mouse	Forebrain	Upregulation	Increases fear memory	Suzuki et al., 2011 [23]
*Crtc1*	Mouse	HPC	Upregulation	Increases fear memory	Sekeres et al., 2012 [24]
	Mouse	HPC, amygdala	Knockdown	Decreases fear memory Decreases CREB-mediated gene transcription	Nonaka et al., 2014 [25]
*Npas*	Mouse	Global	Knockout	Impairs contextual fear conditioning	Ramamoorthi et al., 2011 [29]
	Mouse	CA3	Selective deletion	Hinders memory formation	
*Serinc1, Serinc3*	Mouse	Non-specified	Upregulation	Promotes vesicle membrane fusion during memory consolidation	Chen et al., 2020 [30]
*Khsrp*	Mouse	Global	Knockout	Impairs memory consolidation in the PFC and HPC	Olguin et al., 2022 [31]
*Foxp1*	Mouse	CA1, CA2	Knockout	Impairs spatial memory	Araujo et al., 2017 [32]
*Srf*	Mouse	Global	Knockdown	Impairs the formation of immediate memory	Etkin et al., 2006 [33]
*Mef2*	Mouse	DG, amygdala	Upregulation	Impairs spatial and fear memory formation	Cole et al., 2012 [34]
	Mouse	DG, amygdala	Decrease	Improves memory formation	
*Arc*	Mouse	HPC	Downregulation	Disrupts reconsolidation after memory retrieval	Goltseker et al., 2023 [38]
*Zif268*	Mouse	Forebrain	Upregulation	Enhances the formation of long-term spatial memories	Penke et al., 2014 [41]
*A2bp1*	Drosophila	Global	Inhibition	Mediates suppression of new memory acquisition	Guven-Ozkan et al., 2016 [46]
*Glu2a*	Mouse	HPC	Inhibition	Suppresses the forgetting of episodic memory	Migues et al., 2010 [54]
Bdnf	Mouse	HPC	Knockout	Cue-dependent fear extinction deficits	Patterson et al., 1996 [64]
Calcineurin, N*fat*	Mouse	HPC	Inhibition	Impairs memory extinction	De la Fuente et al., 2011 [68]
NF-κB	Mouse	HPC	Inhibition	Enhances memory extinction	De la Fuente et al., 2011 [68]

**Figure 2:**
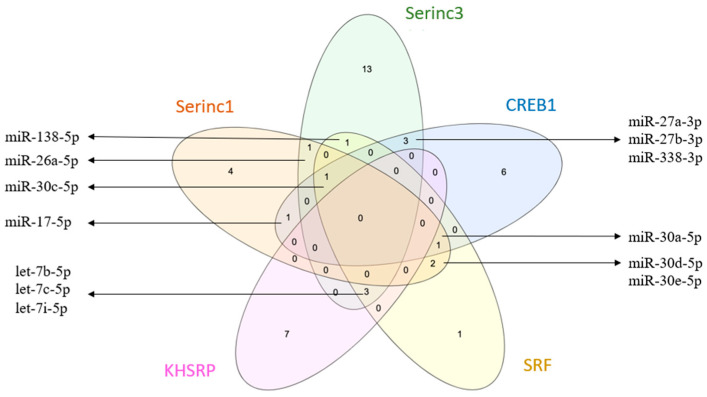
Venn diagram of regulatory miRNAs overlaps from the previous table. Genes that had overlapping regulatory miRNAs were combined in this figure. For example, miR-30a-5p was found to regulate the expression of *SRF*, *CREB1* and SERINC1.

By using an experimentally validated miRNA database, we extracted a list of miRNAs that regulate the expression of genes involved in memory regulation mentioned in this review (Table 2) [77]. For instance, miR-34a-5p has been shown to regulate the expression of gene CRTC1 in human brain tissue. In addition, when overexpressed, CRTC1 is thought to increase fear memory. Additionally, we observed overlaps between the regulatory miRNAs that we represented in a Venn Diagram below (Figure 2).

**Table 2:** Genes discussed in this article and their experimentally validated regulatory miRNAs. The organism and tissue in which it was experimented is also mentioned.

Gene	miRNA	Organism	Tissue	Effect	Reference
*CRTC1*	miR-34a-5p	Human Mouse	Brain	Modulating fear memory	Boudreau, R.L. et al., 2014 [27]
	miR-124-3p				
	miR-181a-5p				
	miR-181b-5p				
	miR-22-3p				
*ARC*	miR-449a-5p	Mouse Human	Brain	Disrupts reconsolidation after memory retrieval	Chi Sung Wook et al., 2010 [51]
	miR-10b-5p				
*KHSRP*	miR-98-5p	Human Mouse	Brain	Modulating memory consolidation	Boudreau, R.L. et al., 2014 [27] Chi Sung Wook et al., 2010 [51]
	let-7(a,b,c,e,f,g,i)-5p				
	let-7(b,c,i)-5p				
	miR-181(a,b)-5p				
*Serinc1*	miR-17-5p	Mouse	Brain	Promotes vesicle membrane fusion during memory consolidation	Chi Sung Wook et al., 2010 [51]
	miR-26a-5p				
	miR-193b-3p				
	miR-30(a,c,d,e)-5p				
	miR-344-3p				
	miR-374b-5p				
	miR-9-5p				
*SERINC3*	miR-16-5p	Mouse Human	Brain	Promotes vesicle membrane fusion during memory consolidation	Chi Sung Wook et al., 2010 [51] Boudreau, R.L. et al., 2014 [27]
	miR-27(a,b)-3p				
	let-7(b,c,i)-5p				
	miR-26a-5p				
	miR-340-5p				
	miR-138-5p				
	miR-425-5p				
	miR-23(a,b)-3p				
	miR-338-3p				
	miR-30(b,c)-5p				
	miR-19(a,b)-3p				
	miR-218-5p				
	miR-128-3p				
	miR-320a				
	miR-125a-5p				
	miR-125-5p				
*CREB1*	miR-338-3p	Human Mouse	Brain	Modulating memory consolidation	Boudreau, R.L. et al., 2014 [27] Chi Sung Wook et al., 2010 [51]
	miR-495-3p				
	miR-153-3p				
	miR-432-5p				
	miR-124-3p				
	miR-17-5p				
	miR-27(a,b)-3p				
	miR-30(a,c)-5p				
	miR-3(d,e)-5p				
*Foxp1*	miR-128-3p	Mouse	Brain	Impairs spatial memory	Gaizka Otaegi et al., 2011 [78]
	miR-17-5p				
*Srf*	miR-22-3p	Mouse	Brain	Impairs the formation of immediate memories	Chi Sung Wook et al., 2010 [51]
	let-7(b,c,i)-5p				
	miR-138-5p				
	miR-30(a,c,d,e)-5p				

## 3. Where Are We Heading?

Memory modulation is a complex process that involves various transcriptional changes occurring at different stages, including acquisition, consolidation, and reconsolidation. We discussed recent research areas that have shed light on the crucial role of transcriptional changes in memory formation, consolidation, reconsolidation, and retrieval. Additionally, we recognized the significance of non-neuronal cells in this process. Furthermore, we have explored how the study of fear memory modulation has provided valuable insights into potential approaches for treating memory-related disorders, such as PTSD. We discussed memory modulation changes from three main aspects: time, location, and interaction. While each of these need to be considered in order to fully understand the intricate mechanisms of memory modulation, none can explain memory modulation regulation by itself. We are showcasing one layer of regulation here, specifically microRNAs, but there are plenty more layers to explore. In conclusion, it is evident that further research is needed in this area to comprehensively grasp the intricate relationship between transcriptional regulation and memory modulation. This research will not only enhance our understanding but also pave the way for the development of more effective treatments for memory-related disorders.

### 3.1. Implications of Transcriptional Regulation of Memory Modulation

This article emphasizes the significance of acquiring a comprehensive understanding of the molecular mechanisms that contribute to psychiatric disorders. By revealing the underlying biological mechanisms, we can formulate more efficient treatments for these conditions. This is especially relevant because traditional treatments for psychiatric disorders, especially PTSD, are often insufficient. This underscores the urgent need for new and more effective interventions. Hence, it is crucial for future research to prioritize the identification of new molecular targets for treatment and the development of innovative therapies. This will enable us to effectively address the needs of individuals who are grappling with psychiatric disorders.

Furthermore, the review highlights that recent technological advancements and methods have made it feasible to utilize transcriptional or gene expression changes for diagnostic and therapeutic applications. Hence, it is crucial for us to maximize the use of these tools in order to gain a deeper understanding of the molecular basis of psychiatric disorders and develop more effective treatments. By harnessing these technologies, we can gain valuable insights into the intricate relationship between genes, environment, and behavior, ultimately leading us to identify new targets for intervention. Ultimately, this can lead to improved patient outcomes and a higher quality of life for individuals living with psychiatric disorders.

### 3.2. Limitations

One major limitation of this review is the lack of extensive research specifically focused on PTSD. Instead, we occasionally had to depend on papers that discuss fear memory and attempt to draw connections to PTSD, but these may not fully capture the complexity of the disorder. Hence, it is essential for future research to focus on working with more comprehensive PTSD models in order to enhance our understanding of the condition.

### 3.3. Future Direction

An interesting future direction in this area of research is to explore the neuronal compositions of recent and remote engrams in greater detail. Future research can specifically focus on identifying the transcriptional changes that occur in recent and remote memories, along with investigating the underlying molecular mechanisms that regulate these changes. By gaining a better understanding of the molecular and cellular processes that govern memory formation and consolidation, we can develop new strategies to enhance memory function and prevent memory loss in psychiatric disorders.

A promising avenue for further analysis is to explore the role of non-neuronal cells, such as glial cells and immune cells, in memory research. While historically memory research has primarily focused on neurons, recent evidence suggests that non-neuronal cells also play a critical role in memory formation, consolidation, and retrieval. Hence, future research could delve into the molecular and cellular mechanisms that contribute to the participation of non-neuronal cells in memory processing. Additionally, exploring their potential as targets for developing novel treatments for psychiatric disorders associated with memory dysfunction would be valuable. By expanding our understanding of the non-neuronal contributions to memory, we can enhance our ability to develop effective interventions that address the complete range of biological processes involved in memory formation and consolidation.

Another direction for future inquiry is to focus on replicating and scaling up studies in order to identify markers associated with memory modulation in psychiatric disorders with stronger evidence. By conducting additional replications, we can enhance our confidence in the reliability of these markers and their potential as targets for therapeutic intervention. Furthermore, future research can delve into modulating these markers to evaluate their influence on memory modulation and ascertain their potential as targets for developing new treatments. By continuing to explore and replicate the findings presented in this review, we can make significant strides in advancing our understanding of the molecular mechanisms underlying memory modulation in neuropsychiatric disorders. This, in turn, will enable us develop more effective treatments for individuals living with these conditions.

## Data Availability

Not applicable.
